# Interaction of INPP5E with ARL13B is essential for its ciliary membrane retention but dispensable for its ciliary entry

**DOI:** 10.1242/bio.057653

**Published:** 2021-01-25

**Authors:** Hantian Qiu, Sayaka Fujisawa, Shohei Nozaki, Yohei Katoh, Kazuhisa Nakayama

**Affiliations:** Department of Physiological Chemistry, Graduate School of Pharmaceutical Sciences, Kyoto University, Sakyo-ku, Kyoto 606-8501, Japan

**Keywords:** INPP5E, ARL13B, Cilia

## Abstract

Compositions of proteins and lipids within cilia and on the ciliary membrane are maintained to be distinct from those of the cytoplasm and plasma membrane, respectively, by the presence of the ciliary gate. INPP5E is a phosphoinositide 5-phosphatase that is localized on the ciliary membrane by anchorage via its C-terminal prenyl moiety. In addition, the ciliary membrane localization of INPP5E is determined by the small GTPase ARL13B. However, it remained unclear as to how ARL13B participates in the localization of INPP5E. We here show that wild-type INPP5E, INPP5E(WT), in *ARL13B*-knockout cells and an INPP5E mutant defective in ARL13B binding, INPP5E(ΔCTS), in control cells were unable to show steady-state localization on the ciliary membrane. However, not only INPP5E(WT) but also INPP5E(ΔCTS) was able to rescue the abnormal localization of ciliary proteins in *INPP5E*-knockout cells. Analysis using the chemically induced dimerization system demonstrated that INPP5E(WT) in *ARL13B*-knockout cells and INPP5E(ΔCTS) in control cells were able to enter cilia, but neither was retained on the ciliary membrane due to the lack of the INPP5E–ARL13B interaction. Thus, our data demonstrate that binding of INPP5E to ARL13B is essential for its steady-state localization on the ciliary membrane but is dispensable for its entry into cilia.

## Abstract (precis)

**Summary:** We here demonstrate that the interaction of INPP5E with ARL13B is crucial for its retention on the ciliary membrane but dispensable for its entry into cilia.

## INTRODUCTION

Primary cilia are sensory organelles for extracellular mechanical stimuli, such as fluid flow, and for signaling molecules, such as the Hedgehog (Hh) morphogen (Bangs and Anderson, 2017; Gigante and Caspary, 2020). Cilia comprise axonemal microtubules, which protrude from the mother centriole-derived basal body, and are surrounded by the ciliary membrane. To achieve their function of receiving specific signals, the composition of proteins and lipids of the ciliary membrane and ciliary interior are distinguished from those of the contiguous plasma membrane and cytoplasm, respectively. This distinction relies on the presence of the ciliary gate, which is composed of transition fibers (TFs) of the basal body and the transition zone (TZ) at the ciliary base (Garcia-Gonzalo and Reiter, 2017; Gonçalves and Pelletier, 2017). The TZ restricts entry and exit of ciliary proteins (Nachury and Mick, 2019), and acts as a diffusion barrier for membrane proteins and lipids between the ciliary and plasma membranes (Jensen and Leroux, 2017), and as a permeability barrier for soluble proteins (Takao and Verhey, 2016). Transport of proteins within cilia and across the ciliary gate is mediated by the intraflagellar transport (IFT) machinery composed of multisubunit complexes (IFT-A, IFT-B, and BBSome) and the kinesin-2 and dynein-2 motor complexes (Nakayama and Katoh, 2020; Taschner and Lorentzen, 2016). In addition to the role of the IFT-A complex and the TULP3 adaptor protein in retrograde ciliary protein trafficking powered by dynein-2, these molecules mediate the import of ciliary membrane proteins across the ciliary gate (Badgandi et al., 2017; Hirano et al., 2017; Kobayashi et al., 2020; Mukhopadhyay et al., 2010; Park et al., 2013). On the other hand, the IFT-B complex mediates anterograde ciliary protein trafficking powered by kinesin-2 and the export of ciliary membrane proteins coupled with the BBSome (Eguether et al., 2014; Lechtreck et al., 2013; Liew et al., 2014; Liu and Lechtreck, 2018; Nozaki et al., 2019; Nozaki et al., 2018; Ye et al., 2018). Owing to the importance of the IFT machinery and the TZ for the integrity of cilia, a broad spectrum of hereditary disorders, collectively referred to as the ciliopathies, arise from mutations in the genes of IFT and TZ components (Braun and Hildebrandt, 2017; Reiter and Leroux, 2017), including Joubert syndrome (JBTS), Meckel syndrome (MKS), nephronophthisis, and Bardet-Biedl syndrome (BBS).

The targeting of lipidated membrane proteins, such as C-terminally prenylated INPP5E, to the ciliary membrane is mediated by a distinct system (Jensen and Leroux, 2017; Stephen and Ismail, 2016). C-terminally prenylated and N-terminally myristoylated membrane proteins are first trapped in the cytosol by PDE6D and UNC119, respectively, both of which are RhoGDI-like solubilizing factors for lipidated proteins (Stephen and Ismail, 2016). The release of bound PDE6D and UNC119 from lipidated proteins is stimulated by allosteric binding of the ARL3 GTPase (Fansa and Wittinghofer, 2016; Fisher et al., 2020; Ismail et al., 2011).

A distinct phosphoinositide distribution is maintained on the ciliary membrane, and a key regulator is INPP5E, which hydrolyzes the 5-phosphate of PtdIns(4,5)P_2_ and PtdIns(3,4,5)P_3_ (Conduit and Vanhaesebroeck, 2020). Particularly, owing to the presence of ciliary INPP5E, PtdIns(4)P is enriched in the ciliary membrane, whereas PtdIns(4,5)P_2_ is limited to the ciliary base (Chávez et al., 2015; Garcia-Gonzalo et al., 2015; Nakatsu, 2015). The physiological relevance of the PtdIns(4)P-rich conditions on the ciliary membrane might be associated with the function of TULP3, which acts as an adaptor connecting the IFT-A complex with ciliary membrane proteins (Badgandi et al., 2017; Mukhopadhyay et al., 2010). As the Tubby domain of TULP3 binds to PtdIns(4,5)P_2_, retrograde ciliary protein trafficking mediated by IFT-A and TULP3 is impaired in the absence of ciliary INPP5E, namely under ciliary PtdIns(4,5)P_2_-rich conditions. Thus, in cells derived from *Inpp5e*-knockout (KO) mice, the aberrant accumulation of GPR161, which is a negative regulator of Hh signaling, was observed (Chávez et al., 2015; Garcia-Gonzalo et al., 2015), and release of extracellular vesicles from the ciliary tip was promoted (Phua et al., 2017). On the other hand, another study indicated that hydrolysis of PtdIns(3,4,5)P_3_ by INPP5E at the ciliary base is important for the convergent regulation of Hh and phosphoinositide signaling (Dyson et al., 2017).

INPP5E has a C-terminal CaaX motif for prenylation, and its localization to the ciliary membrane is therefore under the regulation of ARL3 via PDE6D (Fansa et al., 2016; Humbert et al., 2012). In addition, INPP5E has a ciliary targeting sequence (CTS), F^609^DRELYL^615^, to which another small GTPase, ARL13B, binds (Humbert et al., 2012); namely, ARL13B directly determines the ciliary membrane targeting of INPP5E (Humbert et al., 2012; Nozaki et al., 2017). On the other hand, ARL13B was reported to act as a guanine nucleotide exchange factor (GEF) for ARL3 (Gotthardt et al., 2015; Ivanova et al., 2017; Zhang et al., 2016). Thus, it is also possible that ARL13B indirectly regulates the ciliary targeting of INPP5E via stimulating the ARL3-mediated release of PDE6D from INPP5E (Stephen and Ismail, 2016). In this context, it is notable that mutations in the genes of all the components involved in the ciliary targeting of INPP5E are known to cause JBTS, namely, *INPP5E*/*JBTS1*, *ARL13B*/*JBTS8*, *PDE6D*/*JBTS22*, and *ARL3*/*JBTS35* [Parisi and Glass, 2003 (updated 2017)].

In this study, we therefore analyzed how the ciliary localization of INPP5E is determined. Unexpectedly, a stably expressed INPP5E construct lacking the CTS was able to partially restore the normal localization of ciliary proteins in *INPP5E*-KO cells, even though the steady-state localization of the INPP5E construct to the ciliary membrane was not detectable. We eventually found that an INPP5E mutant lacking the CTS is able to transiently enter cilia but is unable to be retained on the ciliary membrane owing to impaired ARL13B binding.

## RESULTS

### INPP5E-KO and ARL13B-KO cells show similar phenotypes

We previously established *ARL13B*-KO cell lines from human telomerase reverse transcriptase-immortalized retinal pigment epithelial 1 (hTERT-RPE1) cells and analyzed their phenotypes, including the targeting of INPP5E to the ciliary membrane (Nozaki et al., 2017). On the other hand, previous studies on the cellular functions of INPP5E were performed using mouse embryonic fibroblasts (MEFs) from *Inpp5e*-KO mice, although these mice themselves were embryonic lethal (Chávez et al., 2015; Dyson et al., 2017; Garcia-Gonzalo et al., 2015; Phua et al., 2017). To directly compare the effects of the absence of ARL13B and INPP5E in the same cell background, we established *INPP5E*-KO cell lines from hTERT-RPE1 cells.

**Fig. 1.**
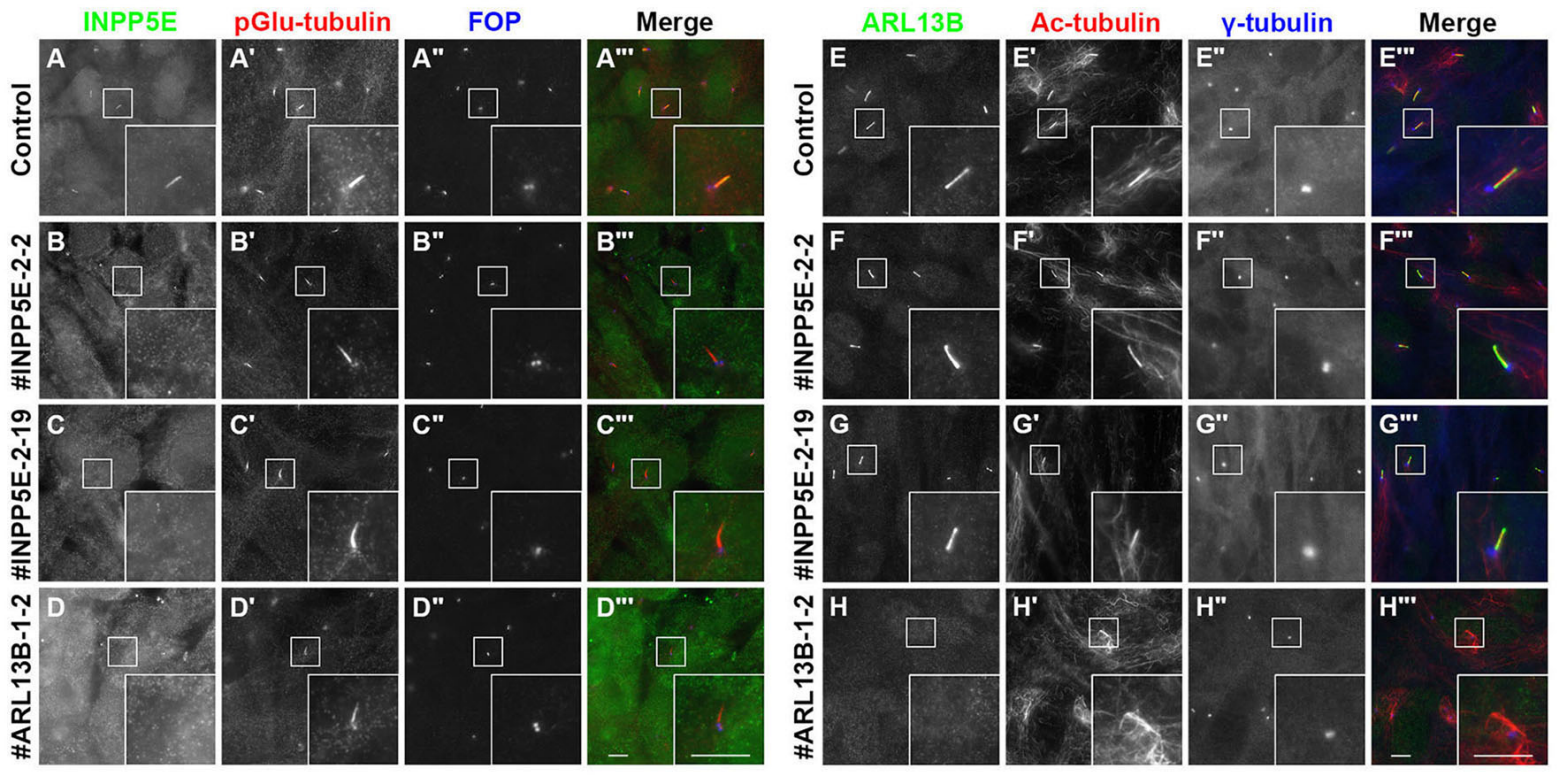
**Localization of INPP5E and ARL13B in *INPP5E*-KO and *ARL13B*-KO cells.** Control RPE1 cells (A,E), the *INPP5E*-KO cell lines #INPP5E-2-2 (B,F) and #INPP5E-2-19 (C,G), and the *ARL13B*-KO cell line #ARL13B-1-2 (D,H), were serum-starved for 24 h and immunostained with a combination of an anti-INPP5E antibody (A–D), the GT335 antibody that recognizes polyglutamylated (pGlu) tubulin (A′–D′), and an anti-FOP antibody (A″–D″), or antibodies against ARL13B (E–H), acetylated α-tubulin (Ac-tubulin) (E′–H′), and γ-tubulin (E″–H″). Insets are 2.5-fold enlarged images of the boxed regions. Scale bars: 5 µm.

Two independent KO cell lines (#INPP5E-2-2 and #INPP5E-2-19) (Fig. S1) were used for the following analyses. In these *INPP5E*-KO cell lines, the ciliary localization of INPP5E was abolished (Fig. 1B,C), whereas ARL13B was retained on the ciliary membrane (Fig. 1F,G). Ciliary INPP5E signals were also absent in *ARL13B*-KO cells (Fig. 1D), as described previously (Nozaki et al., 2017). These observations are consistent with the fact that ciliary membrane localization of INPP5E is dependent on ARL13B.

**Fig. 2.**
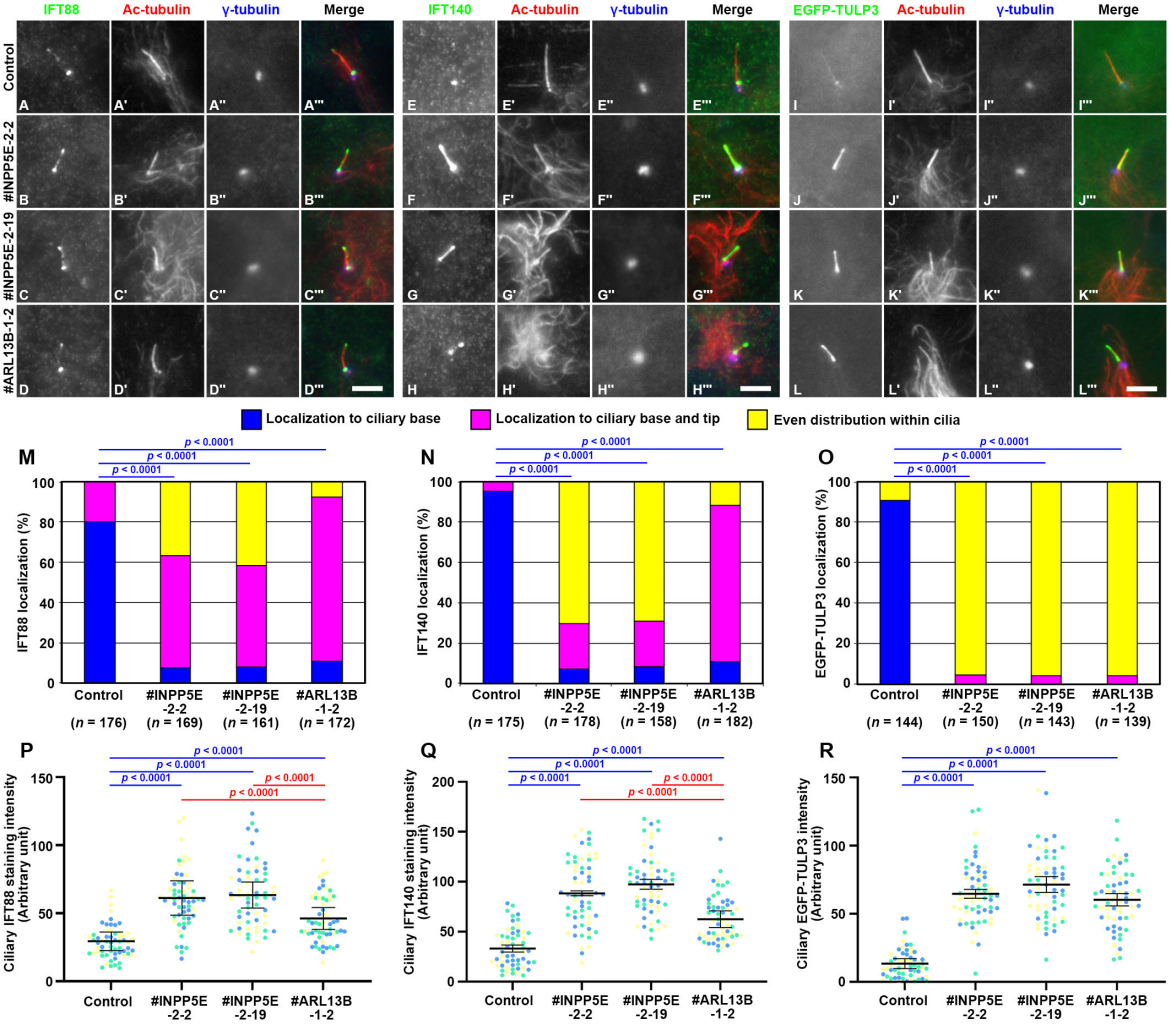
**Accumulation of IFT-A and IFT-B proteins at the ciliary tips of *INPP5E*-KO and *ARL13B*-KO cells.** (A–H) Control RPE1 cells (A,E), the *INPP5E*-KO cell lines #INPP5E-2-2 (B,F) and #INPP5E-2-19 (C,G), and the *ARL13B*-KO cell line #ARL13B-1-2 (D,H), were serum-starved for 24 h and triply immunostained for either IFT88 (A–D) or IFT140 (E–H), Ac-tubulin (A′–H′), and γ-tubulin (A″–H″). (I–L) Control RPE1 cells (I), the *INPP5E*-KO cell lines #INPP5E-2-2 (J) and #INPP5E-2-19 (K), and the *ARL13B*-KO cell line #ARL13B-1-2 (L) stably expressing EGFP-TULP3 were serum-starved for 24 h, and immunostained with antibodies against Ac-tubulin (I′–L′) and γ-tubulin (I″–L″). Scale bars: 5 µm. (M–O) Localization of IFT88 (M), IFT140 (N), and EGFP-TULP3 (O) in individual control, *INPP5E*-KO, and *ARL13B*-KO cells was classified as ‘localization to ciliary base’, ‘localization to ciliary base and tip’, and ‘even distribution throughout cilia’, and the number of cells in each category was counted. The percentages of these populations are expressed as stacked bar graphs. Values are means of three independent experiments, and the total numbers of cells analyzed (*n*) are indicated. In each set of experiments, 53 to 62 cells (M), 49 to 63 cells (N), and 43 to 52 cells (O) were analyzed. Statistical significances were calculated for the ‘base’ category using two-way ANOVA followed by Tukey’s multiple comparison test. (P–R) The relative ciliary staining intensities of IFT88 (P) and IFT140 (Q), and the relative ciliary intensities of EGFP-TULP3 (R) in control, *INPP5E*-KO, and *ARL13B*-KO cells were estimated and expressed as scatter plots. Different colored dots represent three independent experiments (*n*=20×3), horizontal lines are means, and error bars are s.d. Statistical significances among multiple cell lines were calculated using one-way ANOVA followed by Dunnett’s multiple comparison test.

We then analyzed the localization of the IFT-B and IFT-A proteins in *INPP5E*-KO and *ARL13B*-KO cells. In a previous study (Nozaki et al., 2017), we showed that in *ARL13B*-KO cells, there is a tendency of IFT88 (an IFT-B subunit), IFT140 (an IFT-A subunit), and TULP3, which is an adaptor protein connecting the IFT-A complex with PtdIns(4,5)P_2_ on the ciliary membrane, to accumulate at the ciliary tip (Mukhopadhyay et al., 2010). In control RPE1 cells, most IFT88 was found around the ciliary base, with a small proportion at the distal tip (Fig. 2A; also see Fig. 2M); in this context, it is notable that our recent super-resolution imaging study showed the localization of IFT88 at the TFs and in the TZ (Katoh et al., 2020). By contrast, the proportion of IFT88 found at both the ciliary base and tip and the total amount of IFT88 within cilia were significantly increased in *ARL13B*-KO cells (Fig. 2D), as described previously, and in *INPP5E*-KO cells (Fig. 2B,C; also see Fig. 2M,P).

IFT140 was also mainly found at the ciliary base in control RPE1 cells (Fig. 2E), and at both the base and tip in *ARL13B*-KO cells (Fig. 2H; also see Fig. 2N), as described previously. In *INPP5E*-KO cells, IFT140 was more broadly distributed within cilia (Fig. 2F and G; also see Fig. 2N,Q), consistent with a previous study using MEFs from *Inpp5e*-KO mice (Garcia-Gonzalo et al., 2015). EGFP-TULP3 was found mainly around the ciliary base in control cells (Fig. 2I), whereas it was found throughout the entire cilia in *INPP5E*-KO and *ARL13B*-KO cells, resulting in an increase in the total ciliary EGFP-TULP3 level (Fig. 2J–L; also see Fig. 2O,R). These observations are consistent with the notions that the IFT-A adaptor TULP3 binds to PtdIns(4,5)P_2_ (Mukhopadhyay et al., 2010), and that the increased level of PtdIns(4,5)P_2_ caused by INPP5E deficiency on the ciliary membrane results in the ciliary retention of the IFT machinery via the binding of TULP3 to PtdIns(4,5)P_2_.

**Fig. 3.**
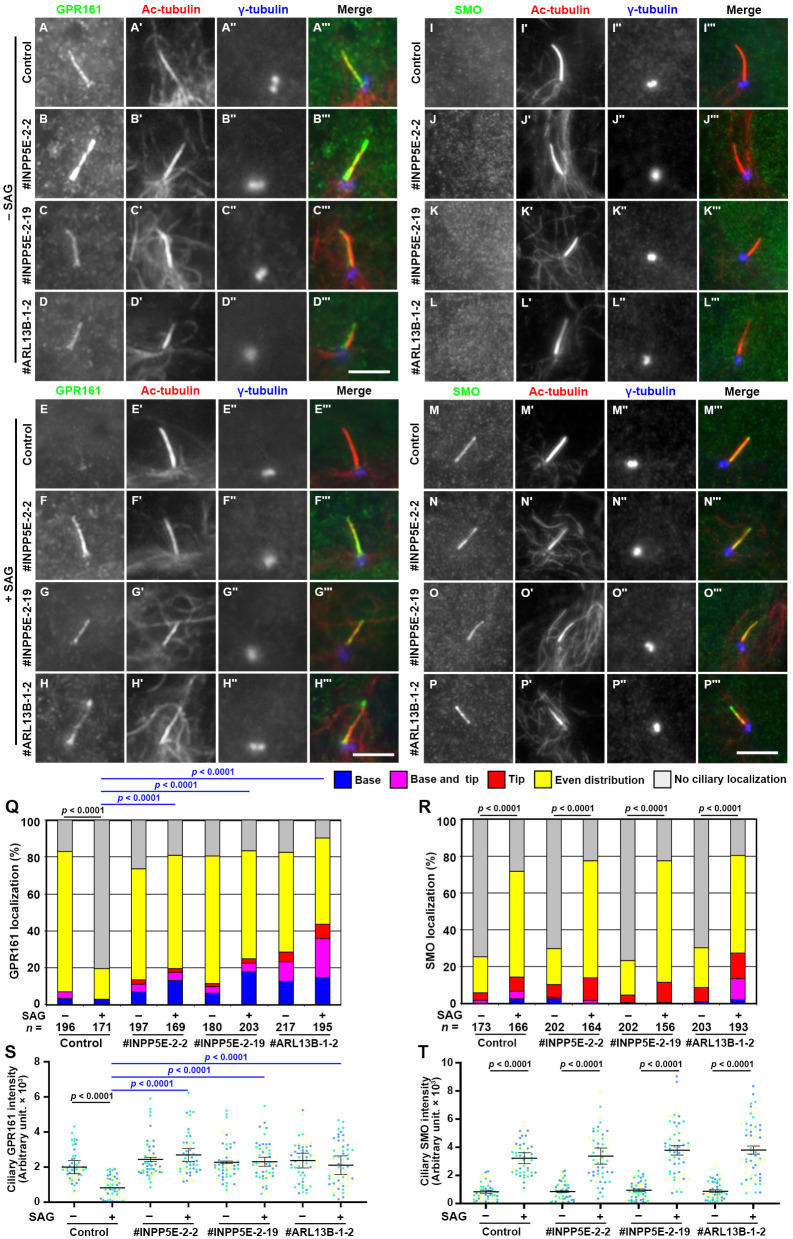
**Accumulation of GPR161 within cilia in *INPP5E*-KO and *ARL13B*-KO cells.** Control RPE1 cells (A,E,I,M), the *INPP5E*-KO cell lines #INPP5E-2-2 (B,F,J,N) and #INPP5E-2-19 (C,G,K,O), and the *ARL13B*-KO cell line #ARL13B-1-2 (D,H,L,P), were serum-starved for 24 h and cultured in the absence (A–D,I–K; −SAG) or presence (E–H,M–P; +SAG) of SAG for a further 24 h, and immunostained with antibodies against either GPR161 (A–H) or SMO (I–P), Ac-tubulin (A′–P′), and γ-tubulin (A″–P″). Scale bars: 5 µm. (Q,R) Localization of GPR161 (Q) and SMO (R) in individual control, *INPP5E*-KO, and *ARL13B*-KO cells was classified as ‘localization to ciliary base’, ‘localization to ciliary base and tip’, ‘even distribution throughout cilia’, ‘localization to ciliary tip’, and no ciliary localization’, and the number of cells in each category was counted. The percentages of these populations are expressed as stacked bar graphs. Values are means of three independent experiments, and the total numbers of cells analyzed (*n*) are indicated. In each set of experiments, 52 to 76 cells (Q) and 52 to 72 cells (R) were analyzed. Statistical significances among multiple cell lines were calculated for the ‘base’ and ‘no localization’ categories using two-way ANOVA followed by Tukey’s multiple comparison test, and those between two groups (−SAG and +SAG) were calculated using Student’s *t*-test. (S,T) Relative ciliary staining intensities of GPR161 (S) and SMO (T) in control, *INPP5E*-KO, and *ARL13B*-KO cells were estimated and expressed as scatter plots. Different colored dots represent three independent experiments (*n*=20×3), horizontal lines are means, and error bars are SD. Statistical significances among multiple cell lines were calculated using one-way ANOVA followed by Dunnett’s multiple comparison test, and those between two groups (−SAG and +SAG) were calculated using Student’s *t*-test.

We then compared the localization of two GPCRs, GPR161 and Smoothened (SMO), in control, *INPP5E*-KO, and *ARL13B*-KO cells. GPR161 and SMO are class A and class F GPCRs, and are negative and positive regulators of Hh signaling, respectively; upon activation of Hh signaling, GPR161 exit cilia, whereas SMO enters cilia (Gigante and Caspary, 2020; Mukhopadhyay and Rohatgi, 2014; Nachury and Mick, 2019). In control RPE1 cells, GPR161 was evenly distributed on the ciliary membrane under basal conditions (Fig. 3A), whereas the majority of GPR161 exited cilia when the cells were stimulated with Smoothened Agonist (SAG) (Fig. 3E; also see Fig. 3Q,S). In striking contrast, GPR161 was retained on the ciliary membrane in *INPP5E*-KO and *ARL13B*-KO cells, even upon stimulation with SAG (compare Fig. 3F–H with B–D; also see Fig. 3Q,S). Thus, as in the absence of ARL13B (Nozaki et al., 2017), the exit of GPR161 from cilia is suppressed upon SAG stimulation in *INPP5E*-KO cells.

On the other hand, in control RPE1 cells, SMO was not found within cilia before SAG treatment (Fig. 3I), and entered cilia upon the stimulation of cells with SAG (Fig. 3M; also see Fig. 3R). In *INPP5E*-KO and *ARL13B*-KO cells, SMO was also absent from cilia under basal conditions (Fig. 3J–L), and entered cilia upon SAG treatment (Fig. 3N–P; also see Fig. 3R,T), similarly to control RPE1 cells. However, the ciliary entry of SMO was not significantly affected by the absence of INPP5E; our data showing that SMO localization was not affected in the absence of INPP5E is consistent with a previous study, probably due to the participation of TULP3 in the ciliary trafficking of class A GPCRs but not that of SMO, which is a class F GPCR (Badgandi et al., 2017; Garcia-Gonzalo et al., 2015).

### Steady-state ciliary localization of INPP5E is not crucial for its role as a modulator of ciliary function

**Fig. 4.**
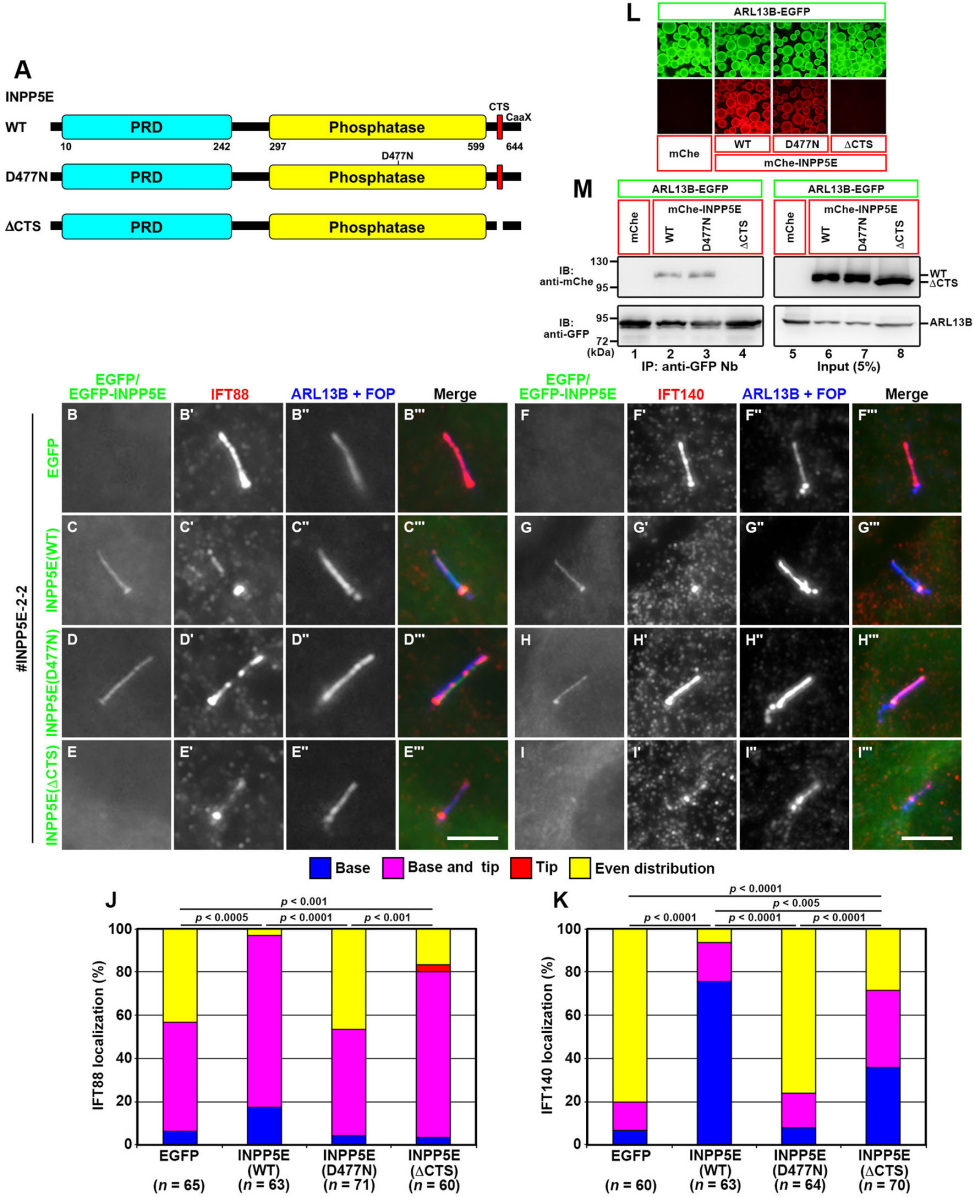
**Rescue of IFT88 and IFT140 localization in *INPP5E*-KO cells upon the stable expression of INPP5E constructs.** (A) Schematic representation of the domain organization of INPP5E and its mutants used in this study. PRD, proline-rich domain. (B–I) The *INPP5E*-KO cell line (#INPP5E-2-2) stably expressing EGFP (B,F), EGFP-fused INPP5E(WT) (C,G), INPP5E(D477N) (D, H), or INPP5E(ΔCTS) (E,I) were serum-starved for 24 h, and immunostained for either IFT88 (B′–E′) or IFT140 (F′–I′) and ARL13B+FOP (B″–I″). Scale bars: 5 µm. (J,K) Localization of IFT88 and IFT140 was analyzed as described in the legend for Fig. 2M,N. In each set of experiments, 18 to 25 cells (J) and 18 to 26 cells (K) were analyzed. Statistical significances were calculated for the ‘even distribution’ category using two-way ANOVA followed by Tukey’s multiple comparison test. (L,M) Lysates prepared from HEK293T cells coexpressing ARL13B-EGFP and mChe, mChe-INPP5E(WT), mChe-INPP5E(D477N), or mChe-INPP5E(ΔCTS) were subjected to the VIP assay using anti-GFP Nb (L), followed by immunoblotting analysis using anti-mChe and anti-GFP antibodies (M). IP, immunoprecipitation; IB immunoblotting.

We then analyzed whether the abnormal phenotypes of *INPP5E*-KO cells can be rescued by the stable expression of INPP5E constructs (see Fig. 4A). As shown in Fig. 4, the stable expression of EGFP-INPP5E(WT) restored the normal localization of IFT88 and IFT140; namely, mainly at the ciliary base (compare panels C and G with B and F, respectively; also see Fig. 4J,K). On the other hand, the expression of EGFP-fused INPP5E(D477N), in which the Asp residue that is crucial for phosphatase activity is substituted to Asn (Bielas et al., 2009; Kong et al., 2006), did not restore the localization of IFT88 or IFT140, even though this mutant itself was able to localize within cilia (Fig. 4D,H), indicating that the phosphatase activity is essential for INPP5E function. Somewhat unexpected was that when EGFP-fused INPP5E(ΔCTS) was expressed in *INPP5E*-KO cells; the localization of IFT88 and IFT140 were partially but significantly restored, although the INPP5E mutant itself was undetectable within cilia (Fig. 4E,I; also see Fig. 4J,K). The INPP5E(ΔCTS) mutant lacks the FDRELYL sequence, to which ARL13B binds (Humbert et al., 2012). We confirmed the binding of ARL13B to INPP5E(WT) but not to the INPP5E(ΔCTS) construct by the visible immunoprecipitation (VIP) assay (Fig. 4L) and by subsequent immunoblotting analysis (Fig. 4M).

**Fig. 5.**
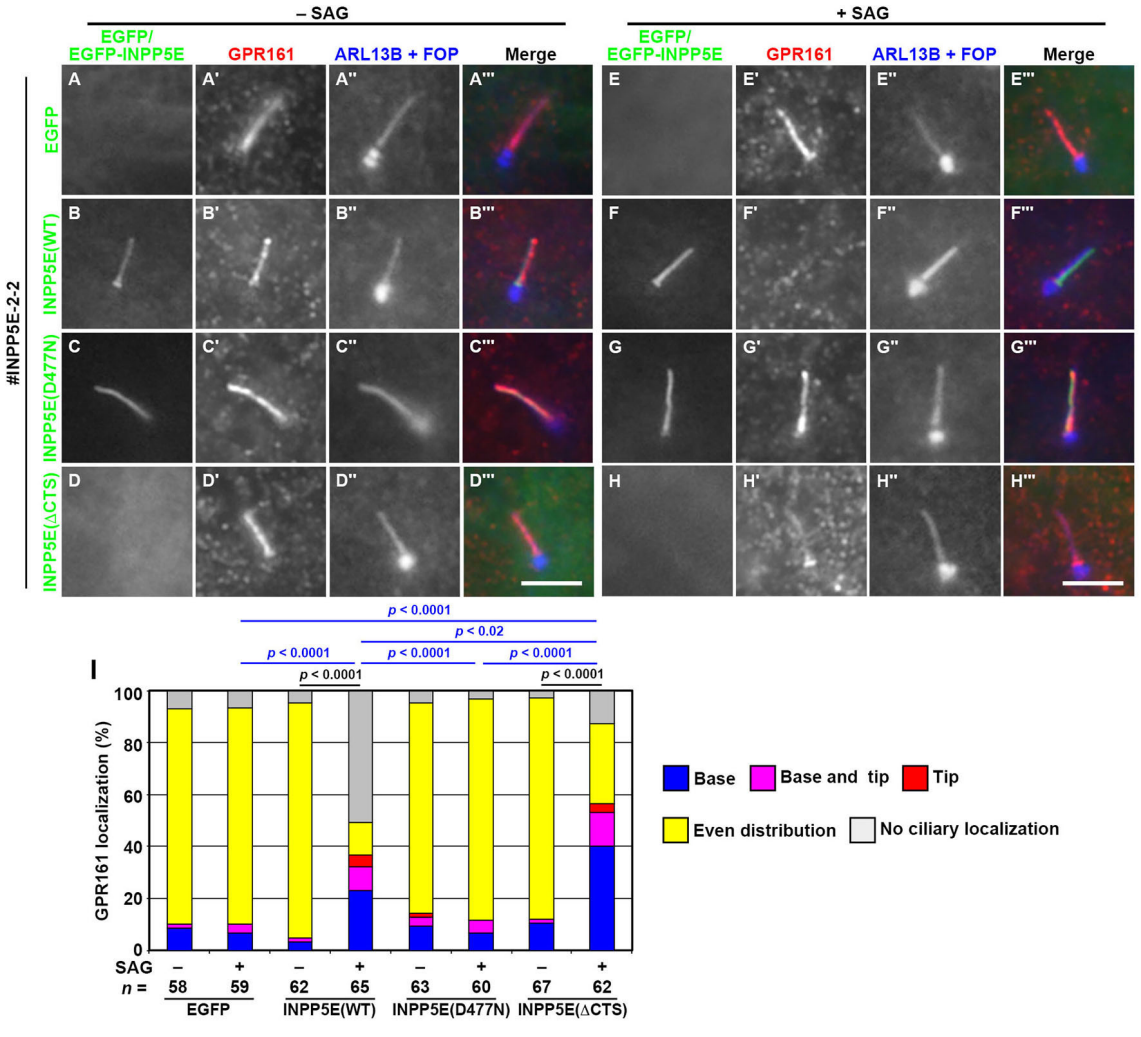
**Rescue of GPR161 localization in *INPP5E*-KO cells upon the stable expression of INPP5E constructs.** The *INPP5E*-KO cell line #INPP5E-2-2 stably expressing EGFP (A,E), EGFP-fused INPP5E(WT) (B,F), INPP5E(D477N) (C,G), or INPP5E(ΔCTS) (D,H) were treated as described in the legend for Fig. 3, and immunostained for GPR161 (A′–H′) and ARL13B+FOP (A″–H″). Scale bars: 5 µm. (I) Localization of GPR161 was analyzed as described in the legend for Fig. 3Q. In each set of experiments, 18 to 23 cells were analyzed. Statistical significances among multiple cell lines were calculated for the ‘base’ and ‘no localization’ categories using two-way ANOVA followed by Tukey’s multiple comparison test, and those between two groups (−SAG and +SAG) were calculated using Student’s *t*-test.

We also analyzed the effects of stable expression of the INPP5E constructs on the GPR161 localization of *INPP5E*-KO cells. The stable expression of EGFP-INPP5E(WT), but not EGFP-INPP5E(D477N), eliminated the retention of GPR161 within cilia after SAG treatment (Fig. 5F,G). On the other hand, the stable expression of EGFP-INPP5E(ΔCTS) partially but significantly rescued the ciliary accumulation of GPR161 upon SAG treatment (Fig. 5H; also see Fig. 5I). Thus, the abnormal phenotypes of *INPP5E*-KO cells appeared to be rescued by the exogenous expression of not only INPP5E(WT) but also the INPP5E construct with compromised ability to target to the ciliary membrane, at least in the steady state.

### The CTS of INPP5E is required for its ciliary retention but is dispensable for its entry into cilia

**Fig. 6.**
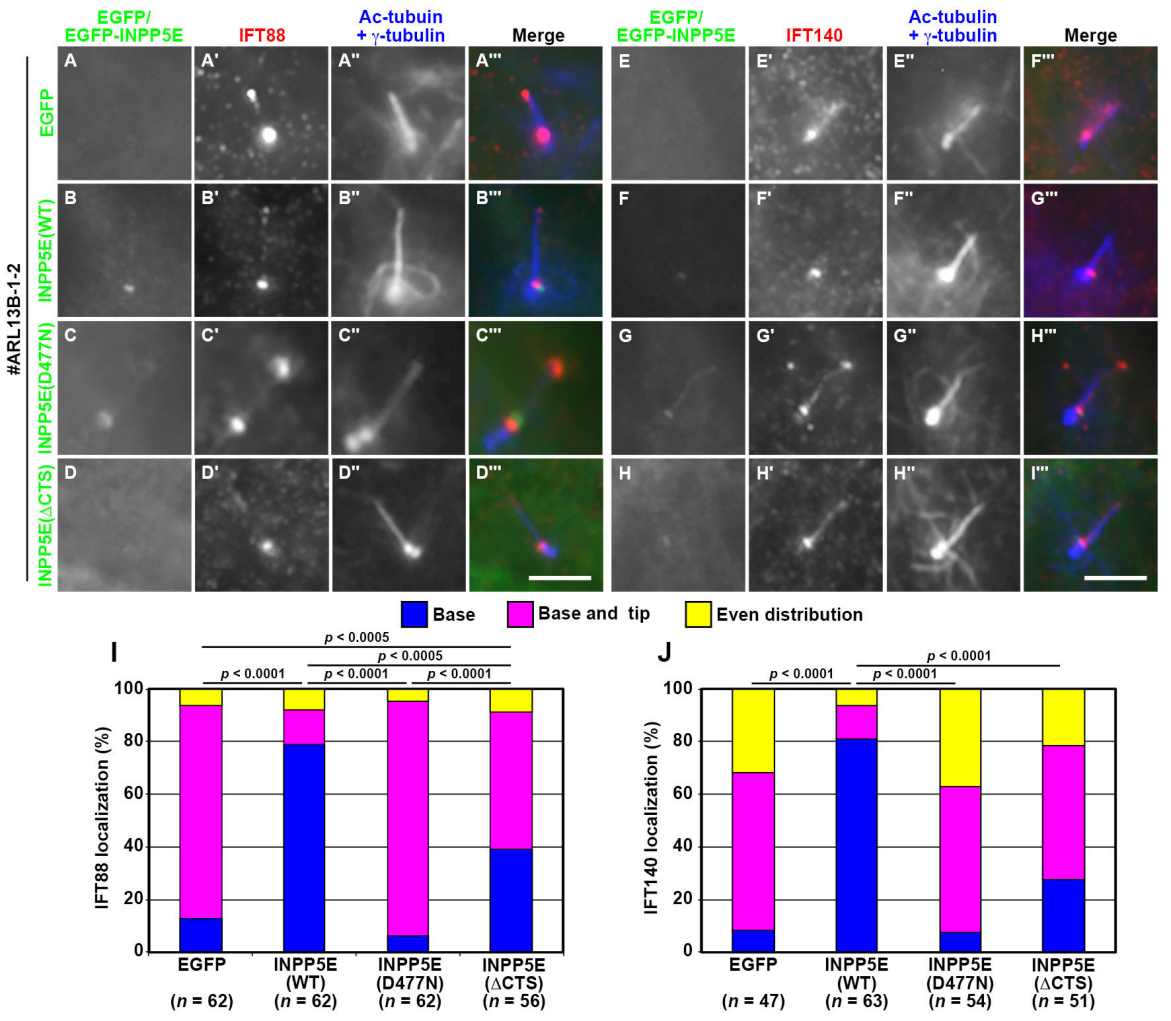
**Rescue of IFT88 and IFT140 localization in *ARL13B*-KO cells upon the stable expression of INPP5E constructs.** The *ARL13B*-KO cell line (#ARL13B-1-2) expressing EGFP (A,E), EGFP-fused INPP5E(WT) (B,F), INPP5E(D477N) (C,G), or INPP5E(ΔCTS) (D,H) were serum-starved for 24 h, and immunostained for either IFT88 (A′–D′) or IFT140 (E′–H′) and Ac-tubulin+γ-tubulin (A″–H″). Scale bars: 5 µm. (I,J) Localization of IFT88 and IFT140 was analyzed as described in the legend for Fig. 2M,N. In each set of experiments, 18 to 21 cells (I) and 15 to 21 cells (J) were analyzed. Statistical significances were calculated for the ‘base and tip’ categories using two-way ANOVA followed by Tukey’s multiple comparison test.

We then investigated the mechanism as to how INPP5E(ΔCTS) partially rescued the defects of *INPP5E*-KO cells, even though the INPP5E construct itself was not detectable within cilia. To this end, we expressed the INPP5E constructs in *ARL13B*-KO cells to analyze whether they were able to rescue the defects of *ARL13B*-KO cells. As expected from the delocalization of endogenous INPP5E in *ARL13B*-KO cells (see Fig. 1D), the ciliary localization of exogenously expressed EGFP-INPP5E(WT) and EGFP-INPP5E(D477N) was barely detectable (Fig. 6B,F and C,G, respectively). However, EGFP-INPP5E(WT) was able to restore the localization of IFT88 and IFT140 predominantly at the ciliary base in *ARL13B*-KO cells (Fig. 6B,F; also see Fig. 6I,J). Furthermore, the normal localization of IFT88 and IFT140 was also significantly restored by the exogenous expression of EGFP-INPP5E(ΔCTS) (Fig. 6D,H; also see Fig. 6I,J).

The absence of INPP5E(ΔCTS) in *INPP5E*-KO cells and INPP5E(WT) in *ARL13B*-KO cells suggests the possibility that INPP5E can modulate ciliary functions outside of cilia. In this context, it is interesting to note the study of Dyson et al. using MEFs from *Inpp5e*-KO mice, in which the authors proposed that INPP5E regulates the molecular organization of the TZ on the basis of their data that TZ proteins were delocalized from the TZ upon treatment of *Inpp5e*-KO MEFs with SAG (Dyson et al., 2017). On the other hand, we recently showed that in KO cells of *MKS1* or *B9D2*, which are components of the MKS module of the TZ (Garcia-Gonzalo and Reiter, 2017), ciliary transmembrane and lipid-anchored membrane proteins, including GPR161, SMO, ARL13B, and INPP5E, are delocalized from cilia irrespective of SAG treatment (Okazaki et al., 2020), and this was due to the disruption of the TZ that acts as a diffusion barrier between the ciliary and plasma membranes.

We therefore analyzed the integrity of the TZ in *INPP5E*-KO and *ARL13B*-KO cells. As shown in Fig. S2A–H, signals for TCTN1, a MKS component of the TZ, were detected at the ciliary base in control RPE1, *INPP5E*-KO, and *ARL13B*-KO cells under both basal (−SAG) and SAG-stimulated (+SAG) conditions [note that we used an anti-TCTN1 antibody from the same commercial source as that used by Dyson et al. (2017)]. We also analyzed the localization of stably expressed EGFP-MKS1, and found that its TZ localization was not changed by SAG treatment in control RPE1, *INPP5E*-KO, and *ARL13B*-KO cells (Fig. S2I–P) [note that the EGFP-MKS1 construct was previously confirmed to rescue the delocalization of ciliary membrane proteins when expressed in *MKS1*-KO cells (Okazaki et al., 2020)]. Thus, our attempts to reproduce the observations of Dyson et al. were unsuccessful. We do not know the exact reason for the apparent discrepancy, but it might be a result of the cells used; i.e. MEFs from *Inpp5e*-KO mice in the study by Dyson et al. and *INPP5E*-KO RPE1 cells in our present study.

On the basis of the results shown in Figs 5 and 6, it is possible that in the absence of the INPP5E–ARL13B interaction, INPP5E is still able to enter cilia but is unable to be retained on the ciliary membrane. To address this possibility, we utilized the chemically inducible dimerization (CID) system (Komatsu et al., 2010; Lin et al., 2013; Takada et al., 2018) to enable trapping of the INPP5E constructs onto the ciliary membrane, controlled by rapamycin (schematically shown in Fig. 7A). We first established control RPE1, *INPP5E*-KO, and *ARL13B*-KO cells stably expressing the SSTR3-mChe-FRB construct, in which mCherry (mChe) and the FK506-binding protein (FKBP)–rapamycin-binding domain (FRB) were fused to the C-terminus of SSTR3, a GPCR constitutively localized on the ciliary membrane (Berbari et al., 2008). Then, N-terminally FKBP-EGFP-fused INPP5E constructs were expressed in the SSTR3-mChe-FRB-expressing cells. In the absence of rapamycin, FKBP-EGFP-INPP5E(ΔCTS) in control RPE1 and *INPP5E*-KO cells and FKBP-EGFP-INPP5E(WT) in *ARL13B*-KO cells were not found within cilia (Fig. 7B,F,H). However, after the addition of rapamycin (final concentration: 200 nM) for 15 min, all the INPP5E constructs were observed within cilia (Fig. 7C,G,I). As a negative control, EGFP-INPP5E(ΔCTS), which lacks the FKBP sequence, did not undergo rapamycin-induced entry into cilia in control RPE1 cells expressing SSTR3-mChe-FRB (Fig. 7D,E). These observations altogether indicate that INPP5E is able to move in and out of cilia across the ciliary gate even in the absence of its binding to ARL13B via its CTS, and even in the absence of ARL13B itself, but is unable to be retained on the ciliary membrane due to the lack of the INPP5E–ARL13B interaction.

**Fig.**
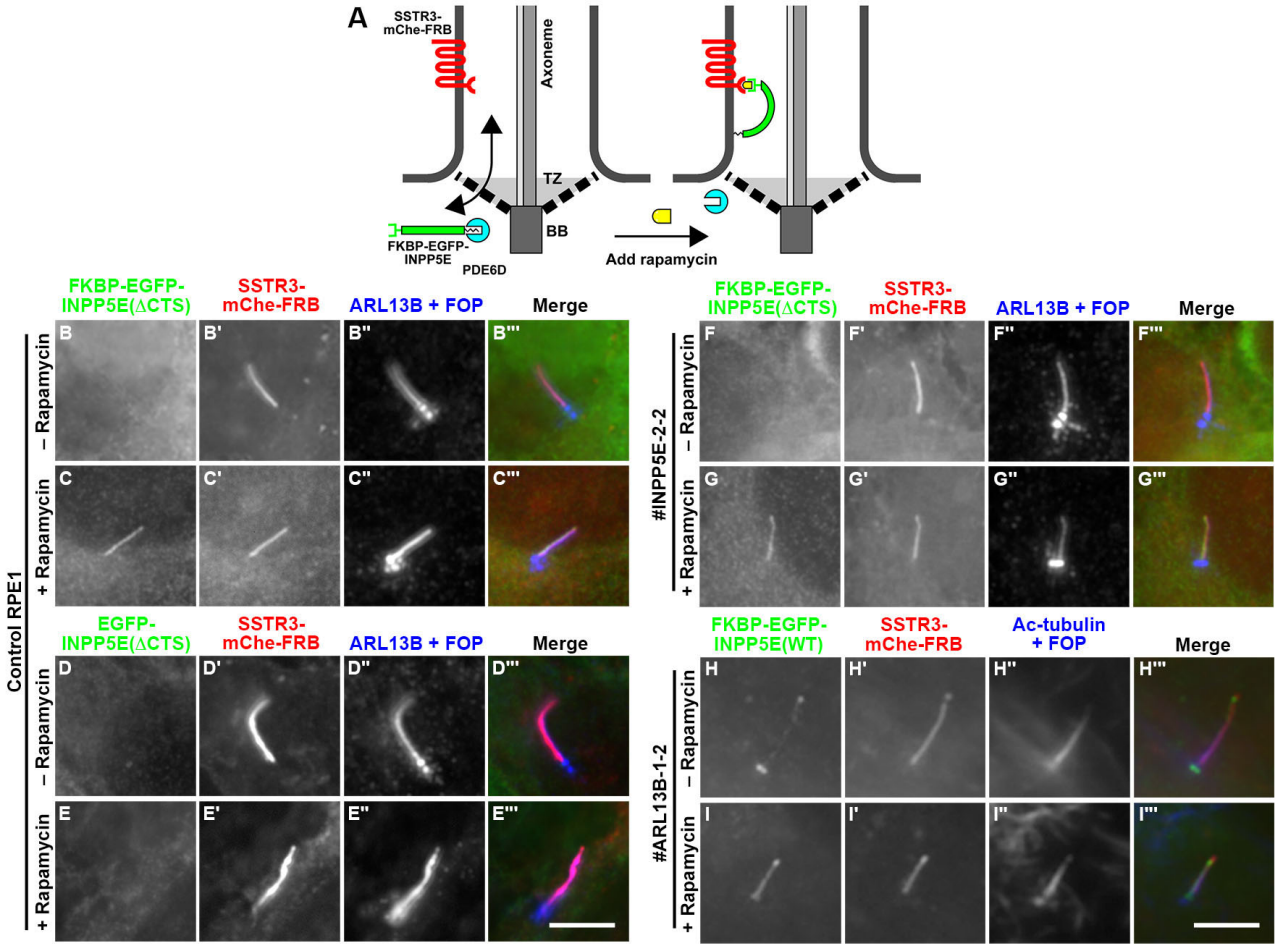
**7. INPP5E–ARL13B interaction is dispensable for INPP5E entry into cilia.** (A) Schematic representation of the design of the CID experiment to enable controlled entry of the INPP5E constructs into cilia. In this model, it is assumed that PDE6D dissociates from INPP5E on the cytosolic side of the TZ. (B–G) Control RPE1 (B–E), *INPP5E*-KO (F,G), and *ARL13B*-KO (H,I) cells stably expressing the SSTR3-mChe-FRB construct were infected with a lentiviral vector for FKBP-EGFP-INPP5E(ΔCTS) (B,C,F,G), EGFP-INPP5E(ΔCTS) (D,E) or FKBP-EGFP-INPP5E(WT) (H,I). The cells were left untreated (B,D,F,H) or treated with 200 nM rapamycin for 15 min (C,E,G,I), immunostained with an anti-GFP antibody (B–I) and anti-ARL13B+anti-FOP antibodies (B″–G″) or anti-Ac-tubulin+anti-FOP antibodies (H″, I″), and observed under a microscope. Scale bars: 5 µm.

## DISCUSSION

Prior to this study, two possible roles of ARL13B in the targeting of INPP5E to the ciliary membrane were proposed, although they are not mutually exclusive. One is that ARL13B determines the ciliary membrane localization of INPP5E by directly interacting with the CTS (Humbert et al., 2012; Nozaki et al., 2017); and the other is that ARL13B indirectly determines the ciliary localization of INPP5E by acting as a GEF for ARL3, which promotes the release of PDE6D from prenylated INPP5E (Gotthardt et al., 2015; Ivanova et al., 2017; Stephen and Ismail, 2016; Zhang et al., 2016).

Our data presented here support the former possibility that the interaction of INPP5E with ARL13B via the CTS is crucial for its retention on the ciliary membrane. Namely, INPP5E(ΔCTS) in control RPE1 cells and INPP5E(WT) in *ARL13B*-KO cells were able to enter cilia but were unable to be retained on the ciliary membrane. In view of the fact that defects in the localization of the components of the IFT machinery and GPR161 were rescued in these cells, the transient entry of exogenously expressed INPP5E molecules into cilia may be sufficient to hydrolyze PtdIns(4,5)P_2_ to PtdIns(4)P in the ciliary membrane. These results are compatible with the fact that the phenotypes of *INPP5E*-KO and *ARL13B*-KO cells closely resemble each other (Figs 2 and 3). In this context, it is important to note the recent study of Gigante et al. showing that knockin mice of Arl13b(V358A), which is an Arl13b variant defective in ciliary localization due to a Val-to-Ala substitution in the VxP ciliary targeting motif (Higginbotham et al., 2012), showed apparently normal Hh signaling, even though ciliary localization of INPP5E was not observed (Gigante et al., 2020). Thus, similarly to INPP5E(ΔCTS), the cilia-excluded Arl13b(V358A) variant might be able to enter cilia but unable to be retained on the ciliary membrane, although it is unknown as to how the VxP motif participates in the ciliary targeting of ARL13B. However, it is also possible that INPP5E(ΔCTS) in control cells and INPP5E(WT) in *ARL13B*-KO cells could function from outside cilia, rather than transient entry into cilia. For example, INPP5E could indirectly affect ciliary protein trafficking through modifying lipid composition on the plasma membrane or on vesicles required for cilia biogenesis.

However, our data does not rule out the latter possibility, which is associated with the important question of where prenylated INPP5E is released from PDE6D, as INPP5E(ΔCTS) retains the ability to undergo prenylation and thereby to be captured by PDE6D in the cytosol. Previous studies showed that INPP5E constructs lacking the C-terminal CaaX motif for prenylation were able to localize to cilia in a PDE6D-independent manner, although their ciliary levels were lower than that of INPP5E(WT) (Humbert et al., 2012; Kösling et al., 2018; Thomas et al., 2014). Thus, without the C-terminal prenylation, INPP5E appears to be able to undergo passage across the ciliary gate, even though the efficiency is low. Once entering cilia, INPP5E molecules without prenylation are likely to be trapped by ARL13B. However, in PDE6D-depleted and *PDE6D*-KO cells, INPP5E or another prenylated protein, RPGR, was not detectable within cilia (Dutta and Seo, 2016; Thomas et al., 2014; Zhang et al., 2019), indicating that for proteins with C-terminal prenylation, PDE6D is crucial for their solubilization in the cytosol (Fansa et al., 2016). As INPP5E constructs without a prenylation site are expected to act as soluble proteins, the soluble and prenylated proteins are likely to use distinct mechanisms to pass the ciliary gate.

If INPP5E enters cilia across the ciliary gate as a complex with PDE6D, the INPP5E–PDE6D complex must permeate the gate by acting as a soluble protein of approximately 85 kDa (∼70 kDa+∼15 kDa). On the other hand, if INPP5E is released from PDE6D and anchored to the lipid bilayer via its prenyl moiety before crossing the ciliary gate, INPP5E is expected to pass the gate by lateral diffusion. Previous studies suggested that the ciliary gate acts as a size-exclusion permeability barrier for soluble proteins; the entry rate decreases as protein size increases, with entry not detectable for proteins greater than 100 kDa (Breslow et al., 2013; Kee et al., 2012; Takao and Verhey, 2016). Another kinetic study using the CID system showed that larger proteins have the potential to enter cilia through the molecular sieve, albeit with reduced kinetics (Lin et al., 2013). On the other hand, the ciliary entry of specific transmembrane proteins, including class A GPCRs and polycystins, across the ciliary gate is known to be mediated by the TULP3 adaptor protein together with the IFT-A complex (Badgandi et al., 2017; Hirano et al., 2017; Kobayashi et al., 2020; Mukhopadhyay et al., 2010; Park et al., 2013). It is likely that TULP3 captures the ciliary localization sequences of these transmembrane proteins in a PtdIns(4,5)P_2_-dependent manner on the plasma membrane side of the ciliary gate, and releases them on the ciliary membrane side, where the PtdIns(4,5)P_2_ level is low owing to the presence of INPP5E (Badgandi et al., 2017).

However, relatively little is known about how lipidated membrane proteins cross the ciliary gate (Jensen and Leroux, 2017). In other words, although lipidated membrane proteins are first trapped by the solubilizing factor PDE6D or UNC119 in the cytosol, it is presently unclear whether these proteins are released from the solubilizing factor with the aid of ARL3 on the plasma membrane or the ciliary membrane side of the gate. If ARL3 is active on the cytosolic side, INPP5E can be released and anchored to the plasma membrane to cross the ciliary gate by lateral diffusion. If ARL3 requires ARL13B for its activation, the INPP5E–PDE6D complex must permeate the ciliary gate in some way, to retrieve PDE6D from INPP5E by activated ARL3 within cilia, and then INPP5E is anchored to the ciliary membrane, where it is retained via its binding to ARL13B. In this context, although our attempts to show the steady-state localization of endogenous or exogenously expressed ARL3 has not been successful to date, ARL3 is expected to readily permeate the molecular sieve of the ciliary gate, considering its small size (Kee et al., 2012; Kösling et al., 2018; Lin et al., 2013).

In conclusion, our present study demonstrates that binding of INPP5E to ARL13B is essential for its retention on the ciliary membrane, but is not necessary for its entry into cilia.

## MATERIALS AND METHODS

### Plasmids, antibodies, and reagents

Expression vectors for INPP5E and its mutants used in this study are listed in Table S1; some of them were constructed in our previous study (Nozaki et al., 2017); the INPP5E and SSTR3 cDNAs were originally provided by Tamotsu Yoshimori (Osaka University) (Hasegawa et al., 2016) and Yumiko Saito (Hiroshima University) (Nagata et al., 2013), respectively. Plasmids containing FKBP and FRB sequences were kind gifts from Takanari Inoue (Johns Hopkins University) (Komatsu et al., 2010). Point and deletion mutants of INPP5E and EGFP-INPP5E with the N-terminal FKBP sequence, and SSTR3-mChe with the C-terminal FRB sequence, were constructed using the SLiCE cloning method (Motohashi, 2015). Packaging plasmids for the production of lentiviral vectors were kind gifts from Peter McPherson (McGill University) (Thomas et al., 2009). Antibodies used in this study are listed in Table S2. SAG, polyethylenimine Max, and rapamycin were purchased from Enzo Life Sciences, Polysciences, and LC Laboratories, respectively.

### VIP assay and immunoblotting analysis

The VIP assay and subsequent immunoblotting analysis were carried out as described previously (Katoh et al., 2015, 2016) with slight modifications (Nishijima et al., 2017), as follows: HEK293T cells expressing EGFP-tagged and mChe-tagged proteins were lysed in HMDEKN cell lysis buffer [10 mM HEPES (pH 7.4), 5 mM MgSO_4_, 1 mM DTT, 0.5 mM EDTA, 25 mM KCl, 0.05% NP-40]. Experimental details of the VIP assay have been described previously (Katoh et al., 2018).

### Establishment of INPP5E-KO cell lines using the CRISPR/Cas9 system

Disruption of the *INPP5E* gene in hTERT-RPE1 cells (American Type Culture Collection, CRL-4000) by the CRISPR/Cas9 system using homology-independent DNA repair was performed as described previously (Katoh et al., 2017) with slight modifications (Okazaki et al., 2020; Tsurumi et al., 2019). Briefly, single-guide RNA (sgRNA) sequences targeting the human *INPP5E* gene (see Table S3) were designed using CRISPOR (Haeussler et al., 2016). Double-stranded oligonucleotides for the target sequence were inserted into the all-in-one sgRNA expression vector peSpCAS9(1.1)-2×sgRNA (Addgene #80768). hTERT-RPE1 cells grown on a 12-well plate were transfected with the sgRNA vector (1 µg) and the donor knockin vector, pDonor-tBFP-NLS-Neo(universal) (0.25 µg; Addgene #80767), using X-tremeGENE9 reagent (Roche Applied Science). After selection of the transfected cells in the presence of G418 (600 µg/ml), sorting of tBFP-positive cells was performed using the SH800S cell sorter (SONY) at the Medical Research Support Center, Graduate School of Medicine, Kyoto University. To confirm disruption of the *INPP5E* gene, genomic DNA extracted from the isolated cells were subjected to PCR using KOD FX Neo DNA polymerase (Toyobo), and three sets of primers (Table S3) to distinguish the following three states of integration of the donor knockin vector: forward integration (Fig. S1A,B), reverse integration (Fig. S1A,C), and no integration with a small indel (Fig. S1A,a). Direct sequencing of the genomic PCR products was performed to confirm the disruption of both alleles of the *INPP5E* gene.

### Preparation of lentiviral vectors and cells stably expressing EGFP-fused INPP5E constructs and SSTR3-mChe-FRB

Lentiviral vectors for the stable expression of INPP5E constructs and SSTR3-mChe-FRB were prepared as described previously (Hirano et al., 2017; Takahashi et al., 2012). Briefly, pRRLsinPPT-EGFP-INPP5E(WT), pRRLsinPPT-EGFP-INPP5E(D477N), pRRLsinPPT-EGFP-INPP5E(ΔCTS), pRRLsinPPT-FKBP-EGFP-INPP5E(WT), pRRLsinPPT-FKBP-EGFP-INPP5E(ΔCTS), or pRRLsinPPT-SSTR3-mChe-FRB was transfected into HEK293T cells using polyethylenimine Max together with the packaging plasmids (pRSV-REV, pMD2.g, and pMDLg/pRRE). Culture medium was replaced 8 h after transfection, and collected at 24, 36, and 48 h after transfection. The medium containing viral particles was passed through a 0.45-µm filter and centrifuged at 32,000× ***g*** at 4°C for 4 h. The pellet was resuspended in Opti-MEM (Invitrogen) and stored at −80°C until use. Cells stably expressing the construct were prepared by the addition of the lentiviral suspension to the culture medium.

### Immunofluorescence analysis

Induction of ciliogenesis and subsequent immunofluorescence analysis of hTERT-RPE1 cells were performed as described previously (Nozaki et al., 2017; Takahashi et al., 2012). The immunostained cells were observed using an Axiovert 200M microscope (Carl Zeiss) or an Axio Observer microscope (Carl Zeiss). For quantification analysis, all images were acquired under the same setting and imported as TIFF files using ImageJ software. A ROI was constructed by drawing a line of 3-point width along the signal of Ac-tubulin or ARL13B within cilia using a segmented line tool. To correct for local background intensity, the ROI was duplicated and set to a nearby region. Statistical analyses were performed using GraphPad Prism8 (Version 8.4.3; GraphPad Software, Inc.).

### CID system

To induce ciliogenesis, control RPE1 cells, and *INPP5E*-KO and *ARL13B*-KO cells stably expressing SSTR3-mChe-FRB, were grown to 100% confluence on coverslips and starved for 24 h in starvation medium [Opti-MEM (Invitrogen) containing 0.2% bovine serum albumin]. FKBP-EGFP-INPP5E(WT) or FKBP-EGFP-INPP5E(ΔCTS) was then expressed by adding the lentiviral suspension to the starvation medium 24 h before cell fixation. After 24 h of starvation, cells were cultured for an additional 15 min in fresh starvation medium containing dimethyl sulfoxide (−rapamycin) or 200 nM rapamycin (+rapamycin). Immunofluorescence analysis was performed as described above.

## Supplementary Material

Supplementary information
