# Supplementary material for: Interaction of INPP5E with ARL13B is essential for its ciliary membrane retention but dispensable for its ciliary entry
Source: Biol Open. 2021 Jan 25;10(1):bio057653. doi: 10.1242/bio.057653 (PMC7860134; doi:10.1242/bio.057653)
Supplement: Supplementary information [file biolopen-10-057653-s1.pdf]

Supplemental materials

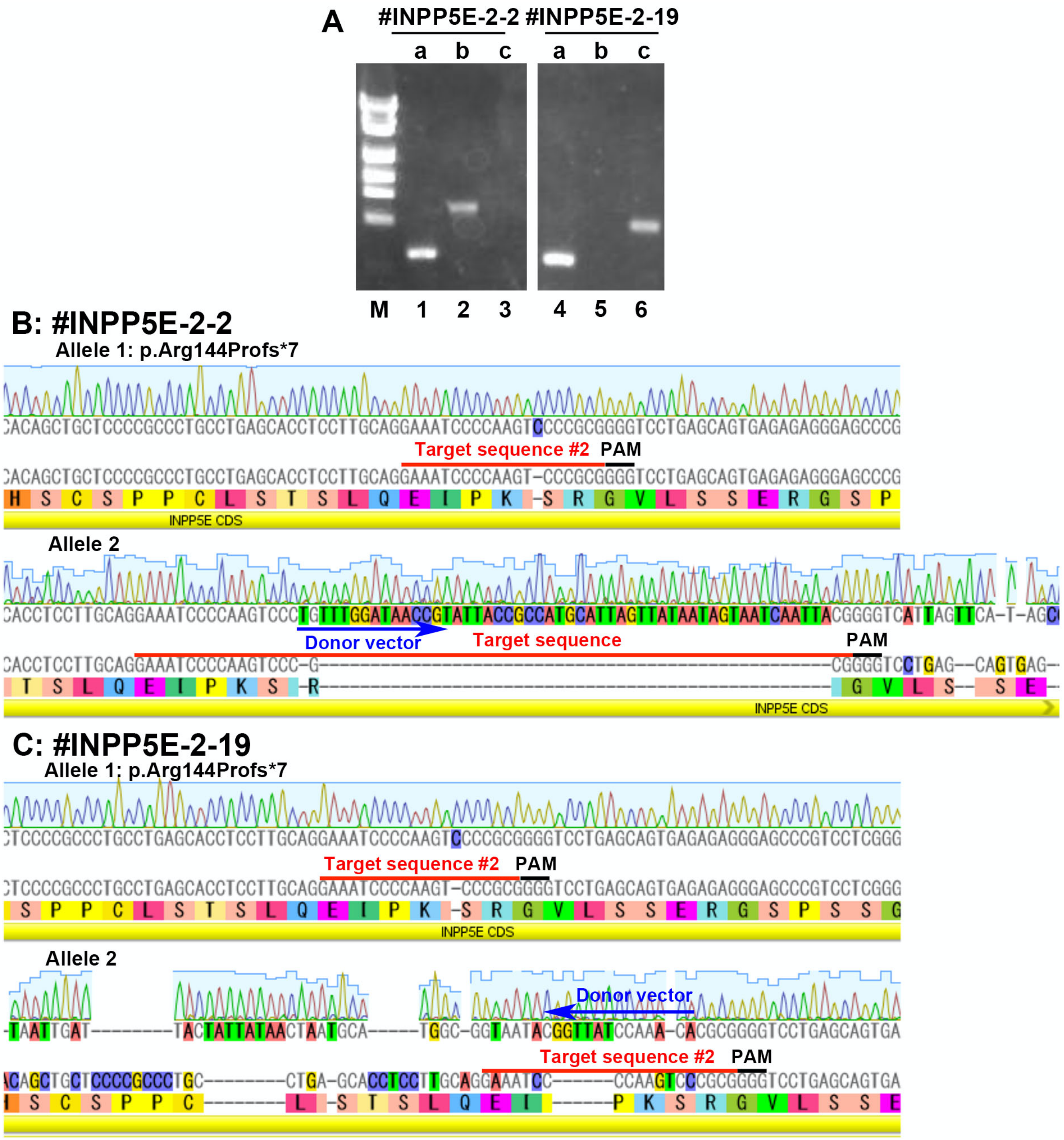

Fig. S1. Genomic PCR and sequence analyses of the *INPP5E*-KO cell lines

(A) Genomic DNA extracted from the *INPP5E*-KO cell lines #INPP5E-2-2 and #INPP5E-2-19 were subjected to PCR using the indicated primer sets (see Table S3) to detect alleles with a small indel or no insertion (a), or with forward (b) or reverse (c) integration of the donor knock-in vector. M, molecular weight marker (PSU1 DNA ladder). (B, C, E, and F) Alignments of allele sequences of the #INPP5E-2-2 (B) and #INPP5E-2-19 (C) cell lines determined by sequencing of the PCR products shown in (A). Red and black lines indicate the target sequence and PAM sequence, respectively, and blue arrows indicate the direction of integration of the donor knock-in vector.

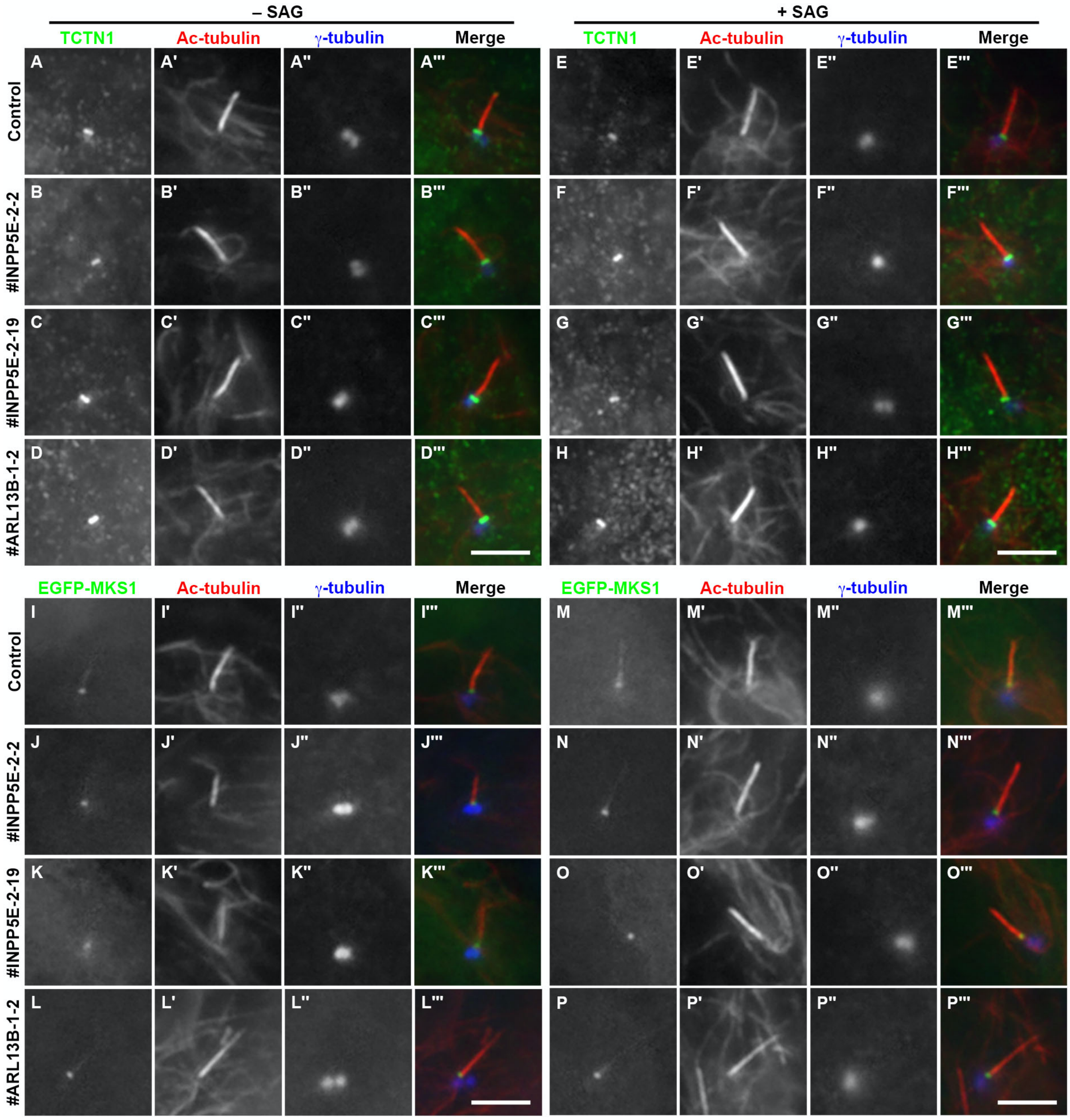

Fig. S2. Integrity of the TZ in *INPP5E*-KO and *ARL13B*-KO cells

(A–H) Control RPE1 cells (A, E), the *INPP5E*-KO cell lines #INPP5E-2-2 (B, F) and #INPP5E-2-19 (C, G), and the *ARL13B*-KO cell line #ARL13B-1-2 (D, H) were serum-starved for 24 h, and cultured in the absence (A–D; –SAG) or presence (E–H; +SAG) of 200 nM SAG for a further 24 h, and immunostained with antibodies against TCTN1 (A–H), Ac-tubulin (A′–H′), and γ-tubulin (A″–H″). (I–P) Control RPE1 cells (I, M), the *INPP5E*-KO cell lines #INPP5E-2-2 (J, N) and #INPP5E-2-19 (K, O), and the *ARL13B*-KO cell line #ARL13B-1-2 (L, P) stably expressing EGFP-MKS1 were serum-starved for 24 h, and cultured in the absence (I–K; –SAG) or presence (M–P; +SAG) of 200 nM SAG for a further 24 h. The cells were immunostained with antibodies against Ac-tubulin (I′–P′) and γ-tubulin (I″–P″). Scale bars, 5 μm.

Biology Open • Supplementary information

Table S1. Plasmids used in this study

Vector	Insert	Reference
pCAG2-EGFP-C	INPP5E	(Nozaki et al., 2017)
pCAG2-mCherry-N	ARL13B	(Nozaki et al., 2017)
pCAG2-EGFP-C	INPP5E(D477N)	This study
pCAG2-EGFP-C	INPP5E(ΔCTS)	This study
pRRLsinPPT-EGFP-C-IRES-Zeo	INPP5E	This study
pRRLsinPPT-EGFP-C-IRES-Zeo	INPP5E(D477N)	This study
pRRLsinPPT-EGFP-C-IRES-Zeo	INPP5E(ΔCTS)	This study
pRRLsinPPT-EGFP-C-IRES-Zeo	TULP3	(Nozaki et al., 2017)
pRRLsinPPT-EGFP-C-IRES-Blast	MKS1	(Okazaki et al., 2020)
pRRLsinPPT-mCh-FRB-N-IRES-Zeo	SSTR3	This study
pRRLsinPPT-FKBP-EGFP-C-IRES-Zeo	INPP5E	This study
pRRLsinPPT-FKBP-EGFP-C-IRES-Zeo	INPP5E(ΔCTS)	This study
pGEX-6P1	Anti-GFP-nanobody	(Katoh et al., 2015)

Table S2. Antibodies used in this study

Antibody	Manufacturer	Clone/catalog number or reference number	Dilution (purpose)
Polyclonal rabbit anti-IFT88	Proteintech	13967-1-AP	1:500 (IF)
Polyclonal rabbit anti-IFT140	Proteintech	17460-1-AP	1:500 (IF)
Polyclonal rabbit anti-ARL13B	Proteintech	17711-1-AP	1:1,000 (IF)
Polyclonal rabbit anti-INPP5E	Proteintech	17797-1-AP	1:500 (IF)
Polyclonal rabbit anti-GPR161	Proteintech	13398-1-AP	1:500 (IF)
Polyclonal rabbit anti-TCTN1	Proteintech	15004-1-AP	1:100 (IF)
Monoclonal mouse anti-ARL13B	Abcam	N295B/66	1:500 (IF)
Monoclonal mouse anti-SMO	Santa Cruz	sc-166685	1:100 (IF)
Monoclonal mouse anti-Ac-tubulin	Sigma-Aldrich	6-11B-1	1:1,000 (IF)
Monoclonal mouse anti-γ-tubulin	Sigma-Aldrich	GTU88	1:500 (IF)
Monoclonal mouse anti-polyglutamylation modification	AdipoGen	GT335	1:500 (IF)
Monoclonal mouse anti-FOP	Abnova	2B1	1:10,000 (IF)
Polyclonal rabbit anti-GFP	Invitrogen	A11122	1:10,000 (IF)
Polyclonal rabbit anti-mCherry	Proteintech	26765-1-AP	1:10,000 (IB)
Monoclonal mouse anti-GFP	Proteintech	66002-1-Ig	1:10,000 (IB)
AlexaFluor-conjugated secondary	Molecular Probes	A11034, A27039, A21244, A11004, A21127, A21240, A21241, A21131, A21242	1:1,000 (IF)
Peroxidase-conjugated secondary	Jackson ImmunoResearch	115-035-166, 111-035-144	1:3,000 (IB)

IF, immunofluorescence; IB, immunoblotting

Biology Open • Supplementary information

Table S3. Oligo DNAs used in this study

Name	Sequence
INPP5E -genome-FW (primer 1)	5'- CGTCCAAGGCGGAGAATCTG-3'
INPP5E -genome-RV (primer 2)	5'- TTGTAGTCTGCAAGATCCGAGTC-3'
pTagBFP-N-RV2 (primer 3)	5'- CGTAGAGGAAGCTAGTAGCCAGG -3'
SLiCE-INPP5E-D477N-S	5'- GGTTTGGAAACTTCAACTTCCGCCTG-3'
SLiCE-INPP5E-D477N-AS	5'- GTTGAAGTTTCCAAACCAGAACACCTC-3'
SLiCE-INPP5E-delCTS-S	5'- AGCTGGGCAAACTAGGAATTAAAAGACGGATTTC-3'
SLiCE-INPP5E-delCTS-AS	5'-TAATTCCTAGTTTGCCAGCTGCCAAC-3'
SLiCE-IRES-FKBP-S	5'-GATGATAAGCTTGCCACAAGCCACCATGGGAGTGCAG-3'
SLiCE-IRES-INPP5E-AS	5'-TGTAATCCAGAGGTTGATTTCAAGAAACGGAGCAGATGG-3'
INPP5E-gRNA#1-S	5'- CACCCCTGGACCCCGATGACATAC-3'
INPP5E-gRNA#1-AS	5'- AAACGTATGTCATCGGGGTCCAGG-3'
INPP5E-gRNA#2-S	5'- CACCGGAAATCCCCAAGTCCCGCG-3'
INPP5E-gRNA#2-AS	5'- AAACCGCGGGACTTGGGGATTTCC-3'

Supplemental references

Katoh, Y., Nozaki, S., Hartanto, D., Miyano, R. and Nakayama, K. (2015). Architectures of multisubunit complexes revealed by a visible immunoprecipitation assay using fluorescent fusion proteins. *J. Cell Sci.* **128**, 2351-2362.

Nozaki, S., Katoh, Y., Terada, M., Michisaka, S., Funabashi, T., Takahashi, S., Kontani, K. and Nakayama, K. (2017). Regulation of ciliary retrograde protein trafficking by the Joubert syndrome proteins ARL13B and INPP5E. *J. Cell Sci.* **130**, 563-576.

Okazaki, M., Kobayashi, T., Chiba, S., Takei, R., Liang, L., Nakayama, K. and Katoh, Y. (2020). Formation of the B9-domain protein complex MKS1–B9D2–B9D1 is essential as a diffusion barrier for ciliary membrane proteins. *Mol. Biol. Cell* **31**, 2259–2268.

Biology Open • Supplementary information
